# Targeting ST3GAL1 to downregulate ligands for the glycoimmune checkpoint Siglec-7 and reverse immune escape in hepatocellular carcinoma

**DOI:** 10.1007/s00262-026-04388-x

**Published:** 2026-04-10

**Authors:** Tan-chi Fan, Tsai-Hsien Hung, Chau-Ting Yeh, Po-Ting Lin, Nai-Chuan Chang, Tzu-Chi Lo, Tsai-Jung Wu, John Yu, Alice L. Yu

**Affiliations:** 1 Institute of Stem Cell and Translational Cancer Research, Chang Gung Memorial Hospital, Linkou Branch, Taoyuan, Taiwan; 2 Liver Research Center, Chang Gung Memorial Hospital, Linkou Branch, Taoyuan, Taiwan; 3 Department of Gastroenterology and Hepatology, Chang Gung Memorial Hospital, Linkou Branch, Taoyuan, Taiwan; 4 College of Medicine, Chang Gung University, Taoyuan, Taiwan; 5 Genomics Research Center, Academia Sinica, Taipei, Taiwan; 6 Department of Pediatrics/Hematology Oncology, University of California in San Diego, San Diego, CA USA

**Keywords:** Sialylation, Sialyltransferase, NK, ADCC, Immunotherapy, Siglec

## Abstract

Sorafenib is the first-line therapy for advanced hepatocellular carcinoma (HCC). However, acquired resistance to sorafenib remains a significant challenge. Previous studies have shown that sorafenib treatment induces the formation of truncated O-glycans in HCC cells, but the relationship between sorafenib-induced glycosylation changes and acquired therapy resistance remains unclear. Primary natural killer (NK) cells, freshly isolated from peripheral blood or following culture and expansion, expressed the glycoimmune checkpoints Siglec-7 and Siglec-9. HCC cells exhibited varying levels of Siglec-7/9 ligands on their surface. Sorafenib-resistant liver cancer cells displayed hypersialylation, leading to increased expression of surface Siglec-7/9 ligands, which conferred protection against NK cell-mediated cytotoxicity. Silencing ST3GAL1 significantly reduced Siglec-7 ligand expression on liver cancer cells, enhancing their susceptibility to NK-mediated cytotoxicity and cetuximab-induced antibody-dependent cellular cytotoxicity (ADCC) in epidermal growth factor receptor (EGFR)-expressing tumor cells. Furthermore, high ST3GAL1 expression correlated with poor clinical outcomes in patients with stage 1-2 HCC. This study highlights the critical role of ST3GAL1 in regulating Siglec-7 ligands to facilitate immune escape from NK cell cytotoxicity. Moreover, its elevated expression is associated with adverse clinical outcomes in HCC. Targeting ST3GAL1 may represent a promising strategy to enhance NK cell-mediated anti-tumor immunity in HCC.

**Supplementary Information:**

The online version contains supplementary material available at 10.1007/s00262-026-04388-x.

## Introduction

Liver cancer, with a five-year survival rate of only 18 %, is the second most lethal malignancy after pancreatic cancer [1]. Hepatocellular carcinoma (HCC) accounts for over 90% of primary liver cancers and is characterized by high VEGF expression, which plays a crucial role in tumor vascularization. Multi-kinase inhibitors targeting VEGFR and PDGFR, such as sorafenib and lenvatinib, have been approved by the FDA as first-line therapeutics for patients with advanced HCC, along with other inhibitors targeting the VEGF/VEGFR signaling pathway, such as regorafenib, cabozantinib, ramucirumab, and bevacizumab. However, patients with HCC often develop acquired resistance to these drugs within six months of treatment [2, 3]. Resistance to anti-VEGFA therapy may be associated with the presence of many VEGF isoforms generated through alternative splicing [4, 5]. As a result, the overall efficacy of anti-angiogenic therapies for advanced HCC remains limited. Therefore, a combination of anti-angiogenic inhibitors and other targeted therapeutic agents is urgently needed to improve therapeutic efficacy.

Glycosylation is the most common post-translational modification in many membrane and secreted proteins, including alpha fetoprotein (AFP), the most widely used marker for HCC detection. Altered glycosylation, which promotes HCC growth, migration, and resistance to anticancer drugs, has been reported in HCC tissues [6]. Sorafenib treatment is associated with increased expression of truncated *O*-glycans in HCC cell lines [7]. However, few studies have investigated the relationship between sorafenib-induced alterations in glycosylation and acquired resistance to therapy.

ST3GAL1 sialyltransferase catalyzes the transfer of sialic acid in an α2,3 linkage to Galβ1-3-GalNAc-Ser/Thr, and thus terminates further chain elongation, except for extension with sialic acids [8]. An altered expression level or activity of this enzyme may lead to changes in the composition and length of* O*-glycans attached to mucin-type proteins. ST3GAL1 is highly expressed in breast cancer, ovarian cancer, colon cancer, prostate cancer, bladder cancer, and glioblastoma [9, 10]. The upregulation of ST3GAL1 has been reported to be significantly correlated with HCC aggressiveness, tumor thrombus, tumor size, and advanced tumor stage [11].

Distinct sialo-glycans act as ligands for sialic acid-binding immunoglobulin-type lectins (Siglecs) receptors expressed on the majority of white blood cells of the immune system to downregulate immune cell signaling [12, 13]. These Siglecs act as glycoimmune checkpoints, with sialo-glycans as their ligands, similar to PD-L1/PD-1, which are currently targeted immune checkpoints for cancer immunotherapy. Siglec-7 and 9 contain an immunoreceptor tyrosine-based inhibition motif (ITIM) to recruit Src homology-2 (SH2) phosphatases and suppress kinase phosphorylation and signaling in immune cells [14]. The major Siglecs expressed in NK cells, dendritic cells and macrophages are Siglec-7 and 9, which function as inhibitory immune checkpoints [15, 16]. One of the Siglect-9 ligands is MUC16 (csMUC16) which is shed into the tumor microenvironment (TME) and detected in the serum of cancer patients as the tumor marker CA125. The binding of csMUC16 to Siglec-9 on immune cells allows cancer cells to evade immune recognition [17]. Antibodies targeting Siglec-7 or 9 reduced the tumor burden in mice [15, 18]. Therefore, blocking Siglecs on immune cells or Siglec-7/9 ligands on cancer cells may show anti-tumor therapeutic potential.

The majority of Siglec-7 targeted glycans on cancer cells are synthesized by ST3GAL1 [19, 20]. Little is known about the role of ST3GAL1 in the ability of Siglec-7 ligands to induce immunosuppression in HCC. In this study, we found that sorafenib treatment induced the expression of sialyltransferases and increased the surface expression of Siglec-7/9 ligands, which protected tumor cells from NK-mediated tumor killing. ST3GAL1 silencing reduced the expression of Siglec-7 ligands in HCC cells. Most importantly, ST3GAL1 silencing significantly enhanced NK-mediated tumor cell death, as well as in the presence of a therapeutic antibody. Therefore, a combination of desialylation and cancer-targeting antibodies may be a novel immunotherapeutic strategy for treating HCC.

## Materials and methods

### Cells, antibodies and reagents

Huh7, and Hep3B cells were kindly provided by Dr. Yang-Hsiang Lin. HA22T (BCRC #60168, RRID:CVCL_7046), HA59T (BCRC #60169, RRID:CVCL_Y018) and HepG2 (BCRC #RM60025, RRID:CVCL_0027) cell lines were purchased from BCRC, Taiwan. All HCC cell lines were cultured in DMEM supplemented with 10% fetal bovine serum (Invitrogen, Carlsbad, CA). The authenticity of the cell lines was confirmed through STR profiling, and they undergo monthly testing for mycoplasma contamination. The reagents were used in this study: DMSO (Sigma, St. Louis, MO), sorafenib (MedChemExpress, Shanghai, China), brefeldin A (Sigma), and permeabilization buffer (Biolegend, San Diego, CA). Recombinant Siglec-Fcs (consisting of an extracellular lectin domain of Siglec and human immunoglobulin G1 hinge-Fc region, with a FLAG tag in between) were kindly provided by Dr. Takashi Angata, Academia Sinica (21). The control (pLAS.Void/pVoid) and *ST3GAL1* short hairpin RNA (shRNA) (TRCN0000231843) plasmids were purchased from the RNAi core (Academia Sinica). The antibodies and lectins used in this study are listed in Supplementary Table S1.

### Establishment of sorafenib-resistant HCC cell lines

To establish acquired sorafenib-resistant cell lines, Huh7 and HepG2 cells were treated with increasing concentrations of sorafenib (starting at 1 uM and increasing the concentration by 10 % at each passage up to 5 uM). Huh7 and HepG2 parental cells were cultured in parallel with the DMSO vehicle control without sorafenib.

### Clinical specimens

Total RNA, from surgically resected tumors, along with relevant clinical and pathological data from 166 patients with American Joint Committee on Cancer HCC stages I to IV, were obtained from the Taiwan Liver Cancer Network (https://tlcn.nhri.edu.tw/). The detailed clinical information and patient characteristics are presented in Supplementary Table S2. All specimens were collected and analyzed in accordance with relevant guidelines and regulations and were approved by the Institutional Review Board of Human Subjects Research Ethics Committee of the Chang Gung Memorial Hospital at Linkou and the Biobank Ethics Committee of the National Health Research Institutes (approval number: 201304758B0C110).

Peripheral blood samples were collected from healthy volunteers. Written consent was obtained from all the participants. The samples were fully encoded and used according to a protocol approved by the Institutional Review Board of the Human Subjects Research Ethics Committee of Chang Gung Memorial Hospital at Linkou, Taoyuan, Taiwan (approval number: 201700831A3). This study was conducted in compliance with the principles of the Declaration of Helsinki.

### Quantitative real-time PCR (qRT-PCR) analysis

Total RNA was isolated using TRIzol reagent (Invitrogen), and cDNA was generated from 1000 ng of total RNA, using a High Capacity cDNA Reverse Transcription Kit (Applied BioSystems/ABI, Foster City, CA). qRT-PCR assays were performed using SYBR Green Real-Time PCR Master Mix or Taqman Master Mix (Applied Biosystems) on an ABI 7500 Detection System (Applied Biosystems). GAPDH was used as an internal control. The qPCR primers used are listed in Supplementary Table S3.

### Siglec-Fc flow cytometry

HCC cells were harvested using 2 mM EDTA/1 % BSA/PBS, washed with PBS, and resuspended in serum free DMEM/1% BSA. The cells were stained with 2 ug/ml Siglec-7or -9-Fc chimera for 30 min at 4 °C. The cells were then washed with PBS and stained with AF488-conjugated goat anti-human IgG for 30 min at 4 °C. The cells were washed with PBS and analyzed using SA3800 (Sony, San Jose, CA) or Accuri C6 Plus (BD, Milpitas, CA). The data were further processed using FlowJo software (v10.9.0).

### Purification of NK cells

Peripheral blood mononuclear cells (PBMC) were harvested from the blood of healthy donors by density gradient centrifugation using Ficoll-Paque (Amersham Biosciences, Uppsala, Sweden), and washed twice with PBS. Peripheral blood natural killer (PBNK) cells were isolated using a human NK cell isolation kit (Miltenyi Biotech, Bergisch Gladbach, Germany), according to the manufacturer’s protocol.

For NK cell expansion, PBMCs were isolated from the peripheral blood of healthy donors by density gradient centrifugation using Ficoll-Paque on day 0. Cells were initially cultured in NK MACS basal medium (Miltenyi Biotec) with AM supplement (Apexcella Biomedical) and 5% human AB serum for 4 days (stage I). From days 4 to 11, cells were cultured in NK MACS basal medium with PM supplement (Apexcella Biomedical) and 5% human AB serum with medium changes every 3–4 days (stage II). From days 11 to 30, NK cells were further expanded in NK MACS basal medium with EM supplement (Apexcella Biomedical) and 5% human AB serum (stage III). Expanded NK cells collected between days 20 and 30 were used for ADCC assays.

### Flow cytometry

PBNK or expanded NK cells were pelleted by centrifugation, washed with PBS and resuspended in serum free DMEM/1% BSA. The cells were labeled with anti-CD56-BV421, anti-CD3-PE, anti-Siglec-7-APC or anti-Siglec-9-FITC for 30 min at 4 °C. The cells were then washed with PBS and analyzed using SA3800 (Sony).

### NK-mediated cell lysis

For NK-mediated cell lysis, 1 x 10^6^ HCC cells/ml were incubated with fluorescent ligand, BATDA (DELFIA EuTDA Cytotoxicity Reagents kit, PerkinElmer, Waltham, MA). One x 10^4^ BATDA-labeled cells were transferred to a round-bottomed 96-well plate and mixed with an antibody (control hIgG or cetuximab) and NK cells. To determine spontaneous release, BATDA-labeled cells were incubated in medium without effector cells. To evaluate the maximal release, complete cell lysis was induced by Triton X-100 (5 % v/v final concentration). Following incubation, the plates were centrifuged and 20 ul of the supernatant from each well was transferred to a flat-bottomed 96-well plate. Finally, 100 ul of Europium solution (PerkinElmer) was added to each well, and the fluorescence of the EuTDA chelates was measured using a time-resolved fluorometer (VICTOR^3^, PerkinElmer). The percentage of specific release was calculated as 100 % (experimental release – spontaneous release)/(maximal release – spontaneous release). All tests were performed in triplicate.

### PBNK-mediated cell lysis

The PBNK-mediated cytotoxicity was assessed using xCELLigence RTCA equipment (Agilent Technologies, San Diego, CA). HCC target cells (8000 cells per well) were plated on 96-well E-plate one day before the ADCC assay. Freshly isolated PBNKs were added to E-plate at an E/T ratio of 2/1 with or without 5 ug/ml cetuximab or control IgG and monitored for an additional 24 hours. The generated data were normalized and analyzed as previously reported [22]. All tests were performed in triplicate.

### Detection of CD107a and IFN-g

Activated NK cell surface CD107a was detected as previously described [23]. In brief, HCC target cells were incubated with 5 ug/ml control IgG or cetuximab for 30 min at RT. PBNKs at an E:T of 1:2 were added and incubated for another 4 h at 37 °C. Then EDTA was added and incubated on ice for 10 min to stop exocytosis. After centrifugation, the cells were stained with an antibody panel containing BV711-anti CD107a, APC-anti CD45 and BV421-anti CD56 antibodies for FACS analysis.

For intracellular IFN-g staining, HCC target cells were incubated with 5 ug/ml control IgG or cetuximab for 30 min at RT. PBNKs at an E:T ratio of 1:2 of were added and incubated for 2h at 37 °C. Brefeldin A (1 ug/10^6 cells) was added and the cells were incubated at 37 °C. Eighteen hours later, the PBNKs were fixed by 4 % PFA, permeabilized with permeabilization buffer, and stained with PE/Cy7-anti IFN-g, APC-anti CD45 and FITC-anti CD56 antibodies for FACS analysis.

### Data source, processing and statistical analysis

The prognostic significance of ST3GAL1 was evaluated using receiver operating characteristic (ROC) area under the curve (AUC) analysis, and the Youden index (sensitivity + specificity − 1) was calculated to determine the optimal cutoff values for high versus low gene expression levels. Survival curves were plotted using the Kaplan-Meier method, with the log-rank test applied for comparison. The Cox proportional-hazards regression model was used to identify the independent prognostic factors. Statistical computations were performed using GraphPad and SPSS V22.0 software.Data are presented as mean ± standard deviation (SD). Statistical analyses were conducted using GraphPad Prism 10. Statistical significance was determined using the two-way ANOVA with multiple comparisons: *P < 0.05, **P < 0.01, ***P < 0.001, ****P < 0.0001.

### Public database analysis of ST3GAL1 expression and survival

Gene expression and clinical data for hepatocellular carcinoma were obtained from the TCGA Liver Hepatocellular Carcinoma cohort (TCGA GDC, 2025) through the cBioPortal database (https://www.cbioportal.org/). ST3GAL1 mRNA expression levels (FPKM z-scores) were extracted for subsequent analysis. The optimal cutoff value for stratifying patients into high and low ST3GAL1 expression groups was determined using ROC curve analysis based on disease-free survival status. The cutoff corresponding to the maximum Youden index was selected as the best threshold. Kaplan–Meier survival curves were generated to evaluate disease-free survival (DFS), and statistical significance between groups was assessed using the log-rank test.

### Kaplan-Meier Plotter database analysis

The association between ST3GAL1 expression and immunotherapy outcomes was analyzed using the Kaplan-Meier Plotter immunotherapy database (https://kmplot.com/analysis/), which integrates gene expression and clinical outcome data from more than 1,400 patients with solid tumors treated with immune checkpoint inhibitors. ST3GAL1 was entered as the gene of interest, and progression-free survival (PFS) was selected as the survival endpoint. Only pretreatment tumor samples were included in the analysis. Patients were stratified into high and low expression groups using the auto-selected best cutoff based on percentile distribution provided by the platform. Subgroup analyses were performed according to treatment with inhibitors targeting PD-1, PD-L1, or CTLA-4 therapies. Kaplan-Meier survival curves, hazard ratios (HRs), and log-rank P values were generated using the database analysis tool. The Kaplan-Meier Plotter immunotherapy database has been described previously [24].

## Results

### Sorafenib resistance induced the expression of ST3GAL1 in Huh7 and HepG2 cells

**Fig. 1:**
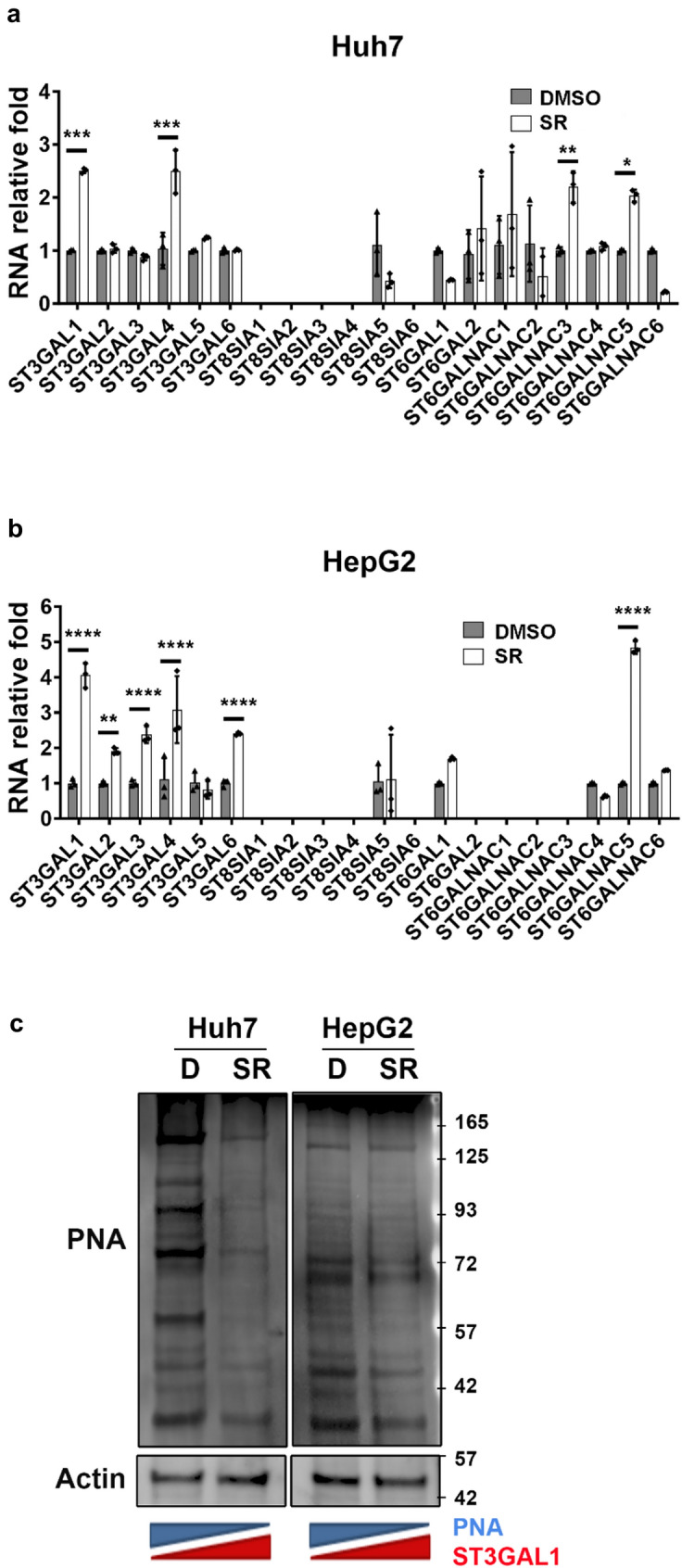
Expression of sialyltransferases in control (DMSO) and sorafenib-resistant **a** Huh7 or **b** HepG2 cells assessed by qPCR analysis. The data are presented as the mean ± standard deviation (SD), based on representative results from three independent experiments (n = 3). Statistical significance was determined using the two-way ANOVA with multiple comparisons: *P < 0.05, **P < 0.01, ***P < 0.001, ****P < 0.0001. **c** Cell extracts from sorafenib-resistant cells were separated and immunoblotted with biotinylated PNA lectin. There was a greater PNA binding signal in the control (DMSO) treated cells. Representative western blot images are shown, derived from three independent experiments. PNA: plant lectin peanut agglutinin; D: DMSO control; SR: sorafenib-resistant cells

HCC cell lines (HCC-SR) with acquired resistance to sorafenib were established in our laboratory. After long-term exposure to sorafenib, the mRNA expression of sialyltransferases was determined by qPCR analysis (Fig. 1a and b). The expression of ST3GAL1, which is involved in the expression of truncated O-glycans, was significantly upregulated in both sorafenib-resistant Huh7 and HepG2 cells. The plant lectin peanut agglutinin (PNA) preferentially recognizes non-sialylated Galβ1-3GalNAc-Ser/Thr core 1 *O*-glycan [25]. When this *O*-glycan is modified by the addition of sialic acid to the α2-3 linkage mediated by ST3GAL1, it generates the trisaccharide, sialyl T (Neu5Acα2-3Galβ1-3GalNAcα1-), which is no longer recognized by PNA lectin. Lectin blotting revealed that major PNA labeling signals were significantly reduced in sorafenib-resistant Huh7 and HepG2 cells (Fig. 1c). These data indicate that long-term exposure to sorafenib induces ST3GAL1 expression.

### ST3GAL1 silencing of HCC cells decreased the cell surface expression of Siglec-7 ligands in HCC cell lines

FACS analysis of a panel of different Siglec-hFc chimeras revealed that pancreatic cancer cell lines express only Siglec-7/9 ligands [19]. However, the expression of Siglec ligands in HCC has not been fully explored. Therefore, the surface expression of Siglec-7/9 ligands on HCC cell lines was assessed by FACS analysis using the Siglec-hFc chimeras. The specificity of Siglec-7/9 hFc chimera binding was confirmed by the loss of signals from Siglec-7/9 Arg120 to Ala mutant. Arg120 is highly conserved within the Siglec family. Mutation of this conserved Arg to Ala within the Siglec family leads to the loss of sialic acid binding [21].

**Fig. 2:**
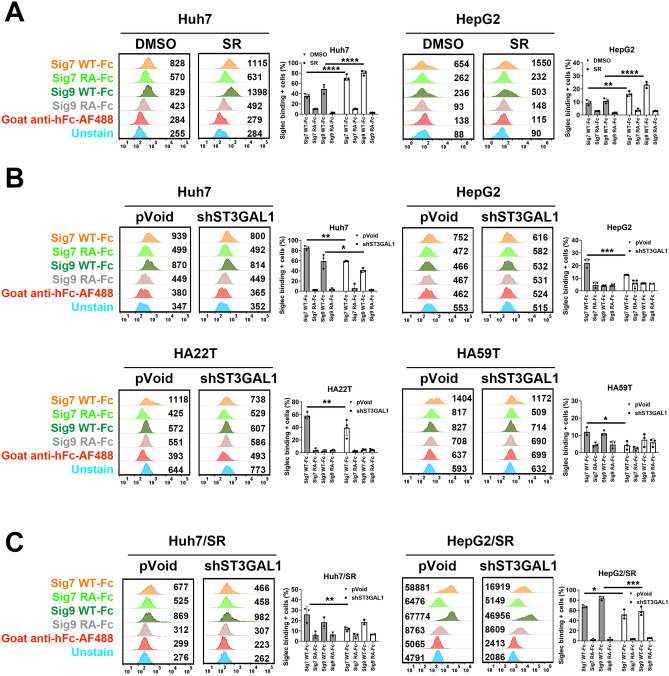
Long-term sorafenib treatment induced the expression of Siglec-7/9 ligands. The cells were stained with recombinant Siglec-Fc chimeras, detected with goat anti-human IgG-AF488 and analyzed by flow cytometry. **a** Sorafenib-resistant cells were stained with recombinant Siglec-Fc chimera and analyzed by flow cytometry. **b** Flow cytometric histograms depicting the expression of Siglec-7 or 9 in control pVoid or *ST3GAL1*-silenced cells by *shST3GAL1* transfection. **c** Control pVoid or *shST3GAL1* transfected sorafenib-resistant cells were stained with recombinant Siglec-Fc chimera and analyzed by flow cytometry. Representative flow histograms from three independent experiments (n=3) are presented, displaying MFI values along with the mean percentages ± SD of Siglec-binding positive cells. Statistical significance was assessed using the two-way ANOVA with multiple comparisons. *P < 0.05, **P < 0.01, ***P < 0.001, ****P < 0.0001. WT: wild type; RA: Siglec-7/9 Arg120 to Ala mutant; SR: sorafenib-resistant; MFI: mean fluorescence intensity

Since long term sorafenib treatment induces multiple sialyltransferases, we investigated whether the surface expression of Siglec-7/9 ligands is altered in sorafenib-resistant cells. FACS analysis using the Siglec-7/9-hFc chimera demonstrated increased binding in both Huh7-SR and HepG2-SR cells compare to control HCC cells (Fig. 2a).

Four HCC cell lines exhibited varying levels of Siglec-7 ligand expression (Fig. 2b). Previous studies have shown that major Siglec-7-binding glycans on pancreatic cancer cells are synthesized by ST3GAL1 (19). qPCR analysis confirmed that *ST3GAL1* was expressed across all tested HCC cell lines (Supplementary Fig. S1a). Silencing *ST3GAL1* by shST3GAL1 transfection markedly reduced the surface expression of Siglec-7 ligands in these HCC cell lines (Fig. 2b). Consistent with previous findings that *ST3GAL1* knockdown diminishes the binding of pancreatic cancer cells to Siglec-7 and Siglec-9, we observed a substantial decrease in Siglec-7 ligand staining following *ST3GAL1* silencing. Unlike Siglec-7 ligands, only Huh7 cells highly expressed Siglec-9 ligands (Fig. 2b). *ST3GAL1* silencing had some effect on Siglec-9 ligand expression.

After *ST3GAL1* silencing, the surface staining of Siglec-7 ligands in HCC-SR cell lines were significantly reduced (Fig. 2c). These data demonstrated that *ST3GAL1* silencing predominantly affects the surface expression of Siglec-7 ligands in HCC cells. Representative overlay histograms for each paired staining are presented in Supplementary Fig. S2. The transcription level of *ST3GAL1* in *shST3GA1* transfected HCC cells was determined by qPCR analysis as shown in Supplementary Fig. S1b.

### Surface expression of Siglec-7 and Siglec-9 on NK cells

**Fig. 3:**
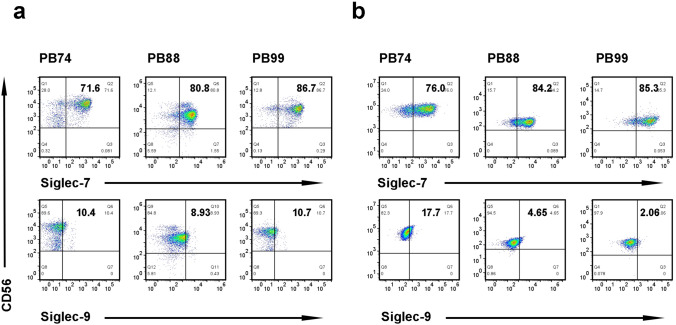
NK cells expressed Siglec-7/9. A representative dot plot showing the expression of Siglec-7/9 on primary NK cells **a** or expanded NK cells **b** determined by flow cytometry using anti-Siglec-7/9 and anti-CD56 antibodies in three healthy donors, respectively

NK cells express different activating and inhibitory surface receptors to distinguish between normal and cancerous cells. It has been shown that the surface of primary NK cells expresses the inhibitory Siglec-7/9 [15, 26]. We investigated the expression of Siglec-7/9 in NK cells (CD56^+^/CD3^−^) expanded from the peripheral blood of healthy donors. As shown in Fig. 3a, PBNK cells predominantly expressed Siglec-7, and some expressed Siglec-9. No significant difference was observed in the number of expanded NK cells compared with the number of primary NK cells (Fig. 3b).

### Increased NK-mediated cytotoxicity and ADCC against HCC tumor cells through *ST3GAL1* silencing

Significant reductions in the function of tumor infiltrating NK cells have been observed in HCC patients [27]. The antitumor activity of NK cells in the absence and presence of cancer-targeting antibodies via ADCC is modulated by the surface expression of inhibitory Siglecs [28, 29]. Since NK cells express high levels of inhibitory Sigle-7/9, NK cell-based therapy in combination with anti-Siglec (ligand) signaling may benefit patients with tumors.

**Fig. 4:**
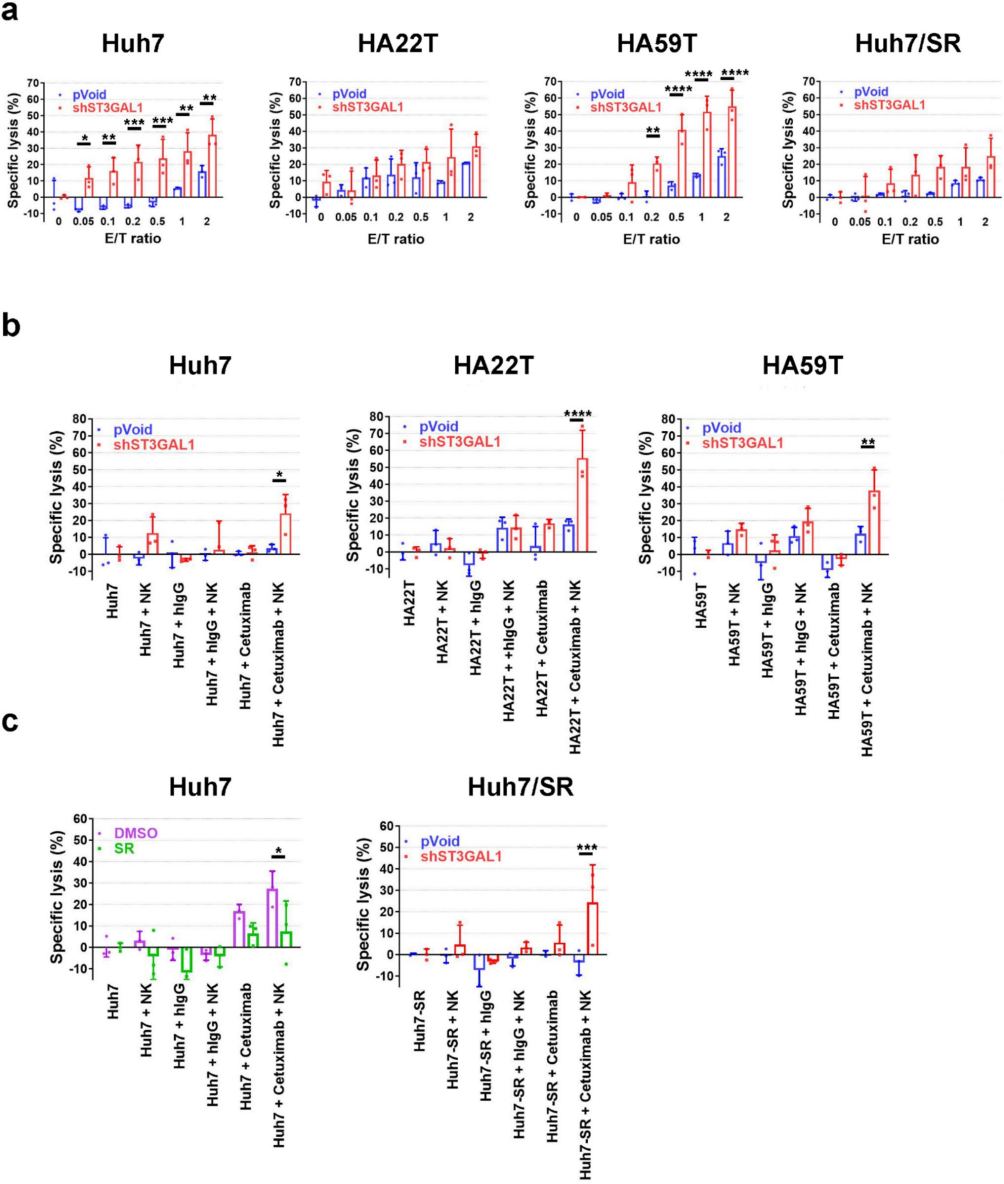
Expanded NK cells mediated cytotoxicity and cetuximab-induced ADCC against HCC cell lines. **a** NK mediated cytotoxicity against control pVoid or sh*ST3GAL1* transfected HCC cells at the indicated E/T ratios. The amount of released BATDA was measured 2 h later. The mean specific lysis of triplicate samples from a single donor is shown. **b**, **c** NK-mediated ADCC against control pVoid or sh*ST3GAL1* HCC cells with or without 0.25 ug/ml control IgG or cetuximab at E/T = 0.2. The amount of released BATDA was measured 2 h later. The mean specific lysis of triplicate samples from a single healthy donor is shown for at least n=3 independent experiments. The bars represent the SDs. SR: sorafenib-resistant; E/T ratio: ratio of effectors to target cells. The data are presented as the mean ± standard deviation, based on representative results from three independent experiments (n = 3). Statistical significance was determined using the two-way ANOVA with multiple comparisons: *P < 0.05, **P < 0.01, ***P < 0.001, ****P < 0.0001

To address whether the inhibitory effect of Siglec-7 ligands on cancer cells is related to the increased expression of ST3GAL1, NK cell-mediated cytotoxicity assays were performed on *ST3GAL1*-silenced HCC cells, achieved through *shST3GA1* transfection. These assays were performed on Huh7, HA22T, and HA59T cells, at different E/T ratios. NK cells were significantly more cytotoxic against all three HCC cell lines transfected with *shST3GAL1* in an E/T ratio-dependent manner (Fig. 4a).

EGFR is overexpressed in 40 to 70 % of conventional HCCs and is correlated with tumor recurrence and extrahepatic metastasis [30, 31]. However, clinical trials of cetuximab, a chimeric monoclonal EGFR IgG1 antibody, showed no antitumor activity in HCC patients [32]. NK cells represent 50 % of the total number of liver lymphocytes and play a vital role in anti-tumor immunity. NK cell function appears to be attenuated in HCC [33], suggesting that reduced cetuximab-mediated ADCC may underlie its lack of efficacy in treating HCC. Approaches to boost the activity of NK cells may help in the NK-mediated eradication of cancer cells either by themselves or by ADCC.

As shown in Supplementary Fig. S3, EGFR was highly expressed in most HCC cell lines, except in HepG2 cells. Consistently, more than 90% of EGFR-expressing HCC cells (including Huh7, HA22T, and HA59T) were stained with cetuximab (Supplementary Fig. S3c), confirming robust EGFR expression. In contrast, HepG2 cells expressed low levels of EGFR, rendering them unsuitable for cetuximab-based ADCC analysis; therefore, HepG2 was were excluded from further functional experiments.

Targeting EGFR with cetuximab alone did not efficiently kill control pVoid transfected EGFR-expressing HCC cells, including Huh7, HA22T and HA59T cells (Fig. 4b). The addition of NK cells also caused little lysis of the control HCC cells. However, in combination with cetuximab, *ST3GAL1* silencing greatly enhanced NK-mediated tumor cell lysis (Fig. 4b). These findings demonstrate that blocking *ST3GAL1* can be used independently or in combination with targeted therapy to enhance the anti-tumor activity of NK cells.

Long-term exposure to sorafenib upregulated the expression of sialyltransferases (Fig. 1) and the surface expression of Siglec-7/9 ligands in HCC cells (Fig. 2a). The cetuximab-mediated ADCC of the expanded NK cells against the sorafenib-resistant Huh7 cells was significantly lower than that of the vehicle DMSO treated Huh7 cells (Fig. 4c). *ST3GAL1* silencing in Huh7/SR cells significantly enhanced NK-mediated cytotoxicity with (Fig. 4c) or without cetuximab (Fig. 4a). These data demonstrated that chronic exposure to sorafenib induced hypersialylation, which protected HCC cells from NK-mediated immune surveillance.

### *ST3GAL1* silencing of HCC cells enhanced Cetuximab mediated PBNK-cell degranulation and cytokine production

**Fig. 5:**
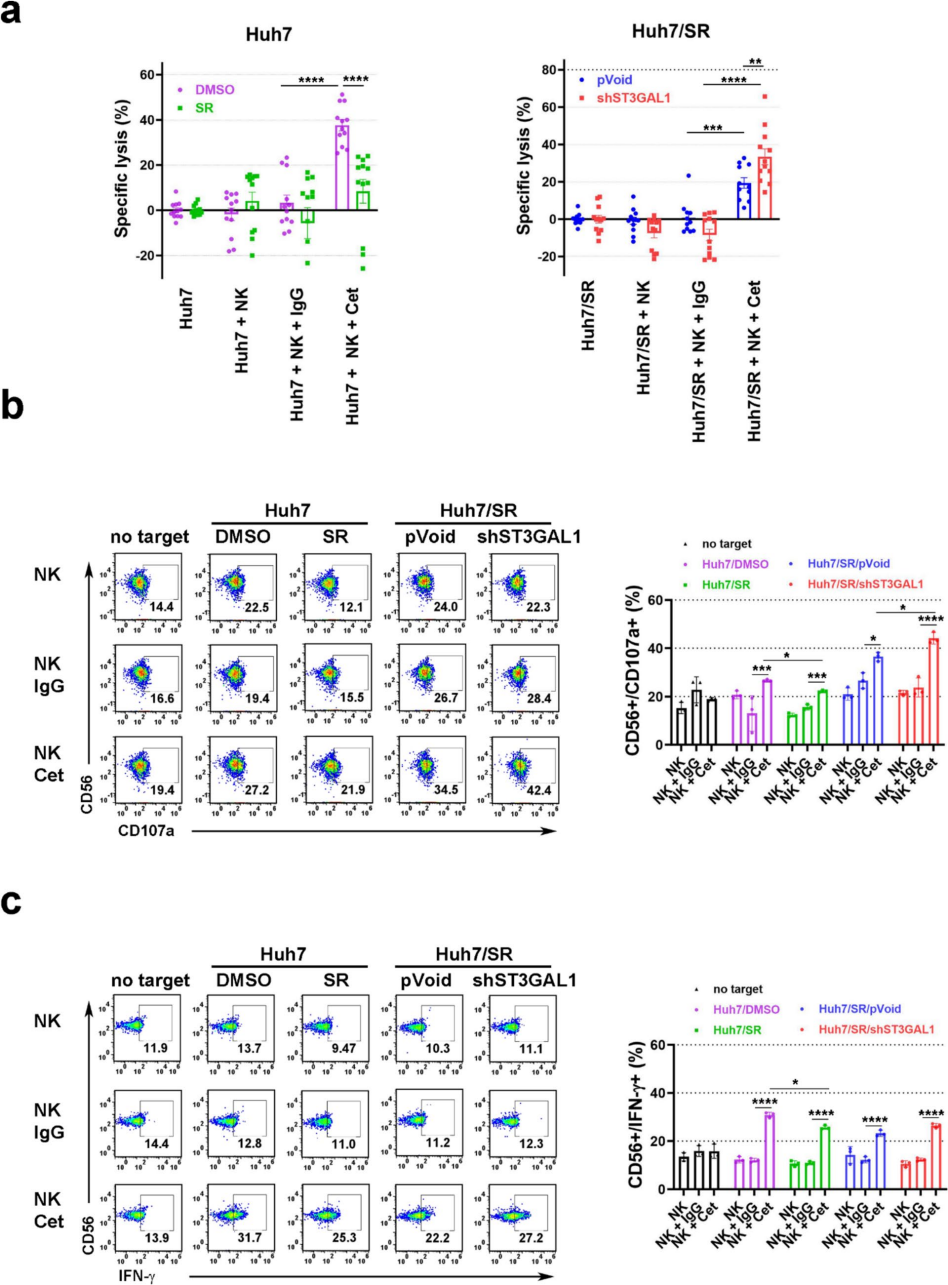
PBNKs-cell degranulation and cytokine production induced by cetuximab-mediated ADCC against Huh7 sorafenib resistant cells. **a** PBNKs mediated cytotoxicity against the indicated cell lines after co-culture for 8 hours at an E/T ratio of 2/1 with or without 5 ug/ml cetuximab or control IgG. The mean specific lysis is shown from n=5 (healthy donors) independent experiments. Bars represent the standard error of measurement (SEM). After incubating PBNKs and HCC cells (E/T ratio of 1/2) with indicated antibodies (5 ug/ml cetuximab or control IgG), PBNK activation was measured by surface staining for CD107a **b** or intracellular IFN-g **c** using flow cytometry. Representative flow plots and mean percentages of PBNKs expressing CD107a or IFN-g are shown for n=5 independent experiments. Bars represent SD, based on representative results from three independent experiments (n = 3). SR: sorafenib-resistant. Cet: cetuximab. Statistical significance was determined using the two-way ANOVA with multiple comparisons: *P < 0.05, **P < 0.01, ***P < 0.001, ****P < 0.0001

Having established the effect of cetuximab on the ADCC of expanded NK cells against HCC cells, the next aim was to extend these findings using freshly isolated PBNK cells against Huh7 sorafenib-resistant cells. PBNKs alone did not show significant cytolytic activity toward Huh7/DMSO or Huh7/SR cells (Fig. 5a). Cetuximab induced significantly greater PBNK-mediated killing in control DMSO-treated Huh7 cells (37.6 %) than in Huh7 sorafenib-resistant cells (8.4 %) (Fig. 5a). Furthermore, *ST3GAL1* silencing significantly enhanced the effect of cetuximab on the ADCC against Huh7 sorafenib-resistant cells (33.4 %) compared with that of pVoid- transfected Huh7 sorafenib-resistant cells (19.4 %) (Fig. 5a). These results are consistent with the ADCC efficacy of expanded NK cells (Fig. 4).

CD107a localized in vesicles in resting NK cells is mobilized to the cell surface upon NK activation. Surface staining for CD107a on activated NK cells has been shown to correlate with the ADCC activity of NK cells [34]. Costimulation of NK cells with cetuximab and cancer cells enhanced IFN-g production [35]. Therefore, we next examined CD107a and IFN-g staining of PBNKs in response to HCC cells and cetuximab. Incubation of PBNKs with cetuximab or control IgG did not significantly increase expression of CD107a (Fig. 5b) or IFN-g (Fig. 5c) on PBNKs. When PBNKs were cocultured with Huh7 cancer cells, the expression of CD107a (Fig. 5b) or IFN-g (Fig. 5c) on PBNKs was not significantly induced. With the addition of cetuximab to PBNKs, CD107a (Fig. 5b) or IFN-g (Fig. 5c) expression was significantly greater in the presence of control Huh7/DMSO cells than in the presence of sorafenib-resistant cells. When *ST3GAL1* was silenced in the Huh7 sorafenib-resistant cells, cetuximab induced a highly significant increases in the percentages of CD107a (Fig. 5b) and IFN-g (Fig. 5c) on PBNKS, as compared to pVoid transfected Huh7 sorafenib-resistant cells. Taken together, these results support that *ST3GAL1* silencing facilitates cetuximab-mediated ADCC against parental or sorafenib-resistant HCC cells.

### Correlation of *ST3GAL1* gene expression with clinicopathological parameters and survival in HCC patients

The clinical relevance of *ST3GAL1* mRNA expression in HCC was assessed using qPCR analysis. To evaluate the predictive potential for disease recurrence, we analyzed the Area Under the Curve (AUC) of the Receiver Operating Characteristic (ROC) curve. The Youden index was used to determine optimal cutoff values for categorizing *ST3GAL1* expression levels as high or low. The relationships between *ST3GAL1* expression and various clinicopathological parameters in HCC patients are presented in Supplementary Fig. S4. Notably, high *ST3GAL1* expression was significantly associated with hepatitis C virus (HCV) infection (*P* = 0.026).

**Fig. 6:**
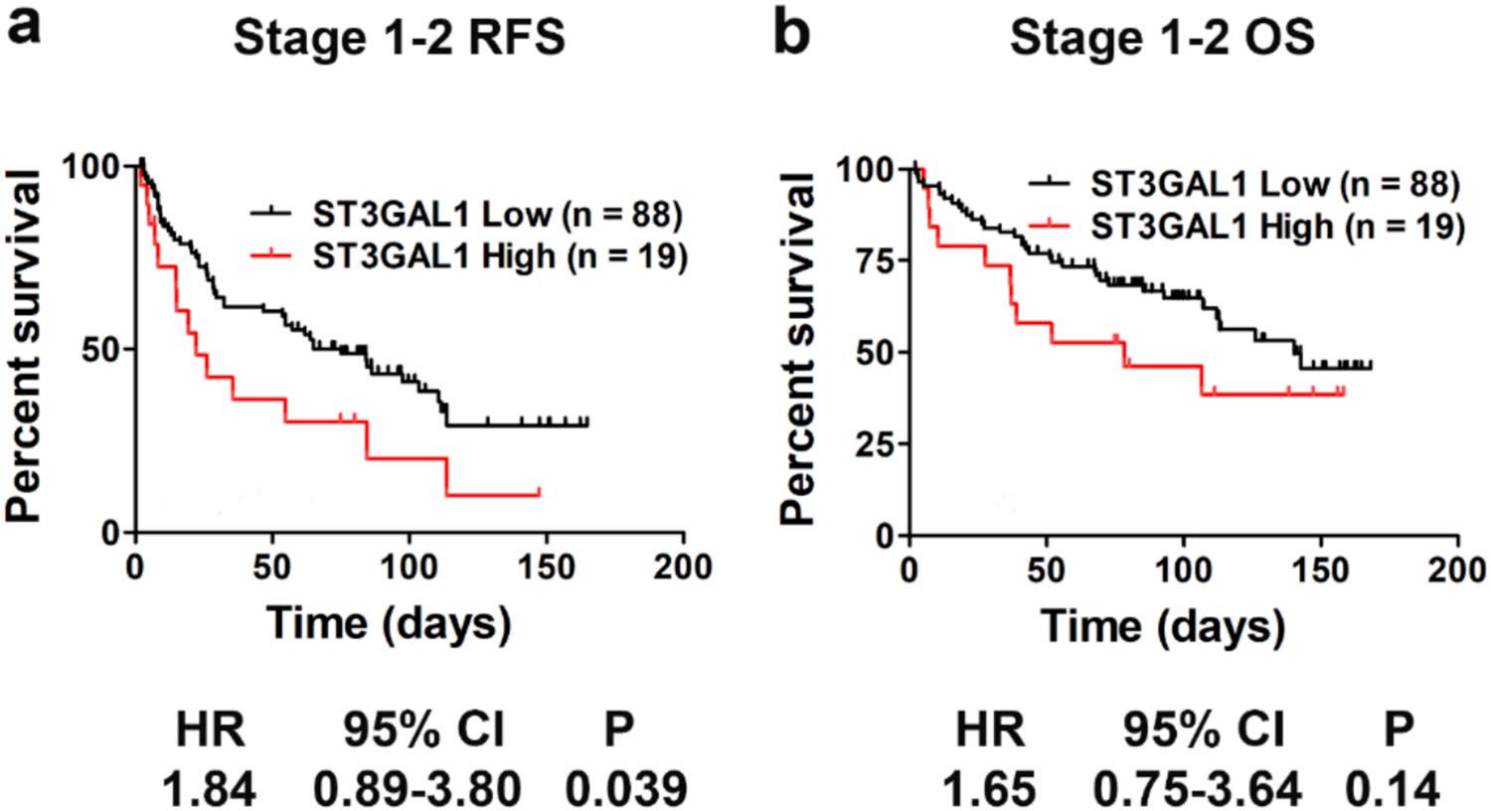
Analysis of survival outcomes in patients with HCC. Kaplan-Meier analyses of relapse free survival (RFS) **a** and overall survival (OS) **b** in patients with stage 1-2 HCC according to *ST3GAL1* expression. A total of 107 patients were included in the analysis, comprising 88 patients in the *ST3GAL1* Low group and 19 patients in the *ST3GAL1* High group. Statistical analyses were conducted using the log-rank test, with Hazard Ratio (HR) values presented below the figure

Kaplan-Meier survival analysis with log-rank testing in patients with stage 1–2 HCC showed that lower ST3GAL1 expression was significantly associated with improved relapse-free survival (RFS) compared with higher ST3GAL1 expression (*P* = 0.039; Fig. 6), although there is a trend for better overall survival but it did not reach statistical significance. A similar finding was observed in the TCGA Liver Hepatocellular Carcinoma cohort (TCGA GDC, 2025) accessed through the cBioPortal database (https://www.cbioportal.org/). Higher *ST3GAL1* expression was associated with worse disease-free survival (DFS) in all stages (*P* = 0.03, Supplementary Fig. S5a) and early stage (*P* = 0.03, Supplementary Fig. S5b). Taken together, these findings support the conclusion that elevated *ST3GAL1* mRNA expression may serve as a predictive biomarker for tumor recurrence, particularly in early-stage HCC.

### High* ST3GAL1* expression correlates with poor clinical response to immune checkpoint blockade

To assess the clinical relevance of ST3GAL1 in immunotherapy response, we analyzed a publicly available immunotherapy cohort from the Kaplan-Meier Plotter database (https://kmplot.com/analysis/). High pretreatment *ST3GAL1* expression was significantly associated with poorer progression-free survival (PFS) in patients treated with immune checkpoint inhibitors targeting PD-1 (*P* = 6.5 × 10^−11^), PD-L1 (*P* = 0.04), or CTLA-4 (*P* = 0.019) in a pan-cancer cohort (Supplementary Fig. S6).

These clinical findings are consistent with our *in vitro* and functional data, supporting the notion that ST3GAL1-mediated hypersialylation contributes to resistance to antibody-based immunotherapy. Together, these results provide a biological rationale for future precision medicine integrating ST3GAL1 expression, and tumor glycosylation status, as potential predictors of immunotherapy efficacy.

## Discussion

The immune checkpoint inhibitor (ICI), atezolizumab (an anti-PD-L1 antibody), in combination with bevacizumab (an anti-VEGFA antibody), was approved by the FDA for the treatment of HCC in 2020. In patients with unresectable HCC, atezolizumab combined with bevacizumab resulted in better overall and progression-free survival outcomes than sorafenib alone [36]. However, the median progression-free survival is only 6.8 months. As human HCC is genetically heterogeneous, the development of novel strategies and/or synergistic combinations to target various mechanisms of resistance is urgently needed.

NK cells account for 25–50% of liver lymphocytes and 10% of lung lymphocytes, suggesting that NK cells may play an important role in tumor killing as the first line of immunity in patients with HCC and lung cancer [37]. The number of NK cells in the peripheral blood of patients with HCC is positively correlated with patient survival and prognosis [38]. Compared with those isolated from healthy donors, NK cells isolated from patients with HCC exhibit defects in lytic function and cytokine secretion [39]. HCC has been classified into inflamed and non-inflamed classes based on inflammatory signatures that predict responses to ICI therapy in patients with advanced HCC. Reduced immune infiltration and downregulated expression of essential NK cell activators, such as *Granzyme B*, *IFN-r* and *NKG2D*, were found in non-inflamed class (~63 % of HCC patients), suggesting an immunosuppressive microenvironment in HCC tissues [40]. The immunosurveillance activity of NK cells is controlled by the balance between activating/inhibitory receptors and their ligands in the TME. NK cells or macrophages can be activated in the presence of therapeutic antibodies to elicit the release of cytotoxic factors that kill tumor cells.

Our qPCR analysis showed that sorafenib resistance induced transcriptional upregulation of PD-L1 and downregulation of VEGFA (Supplementary Fig. S7). The increased expression of PD-L1 in sorafenib-resistant cells may contribute to immune evasion from T-cell-mediated immunity. However, ST3GAL1 knockdown in sorafenib-resistant cells did not significantly affect the expression levels of PD-L1, VEGFA, or EGFR (Supplementary Fig. S3 and S7). On the other hand, ST3GAL1 knockdown markedly enhanced the susceptibility of tumor cells to NK-cell-mediated cytotoxicity and cetuximab-mediated ADCC. We speculate that this increased sensitivity is associated with the downregulation of Siglec-7 ligands on tumor cells, thereby reducing inhibitory signaling to NK cells. This mechanism represents an immune regulatory pathway distinct from current immunotherapeutic strategies targeting PD-L1.

**Fig. 7:**
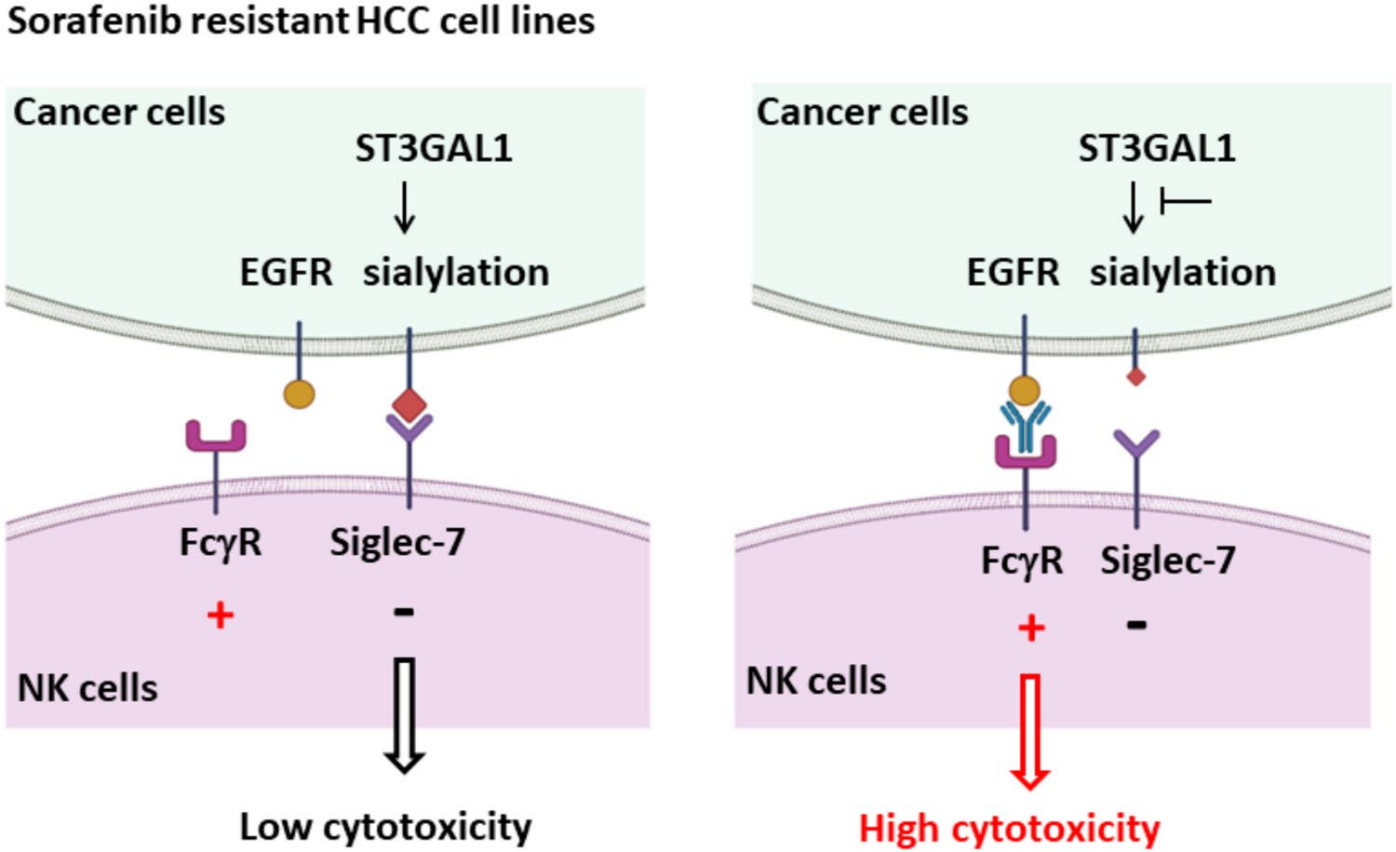
Proposed schema illustrating the role of *ST3GAL1* silencing in enhancing the cytotoxic ability of NK cells against HCC cells

In this study, we illustrated how *ST3GAL1* silencing in HCC cells enhanced the cytotoxic ability of NK cells as depicted in Fig. 7. Siglecs act as immune checkpoints through interactions with sialic acids on cell surface proteins or lipids [13]. Sialylated glycans protect tumor cells from NK-mediated cytotoxicity through interactions with Siglec family members on NK cells [13, 29]. Siglec-7 is a pan-human NK cell marker, whereas Siglec-9 is expressed in CD56^dim^ CD16^+^ NK cells [28]. The expression of Siglec-7 in tumor-infiltrating lymphocytes in colorectal cancer tissues has been shown to be significantly greater in patients with poor prognosis [41]. Recently, Bordoloi *et al* investigated targeting Siglec-7 directly to modulate immune function for ovarian cancer therapy [42]. The addition of anti-PD-1 antibody to a coculture of ovarian cancer cells with PBMCs/NKs slightly improved the PBMC/NKs-mediated killing of ovarian cancer lines. The combination of anti-Siglec-7 and anti-PD-1 further improved ovarian cancer killing *in vitro*. Combination treatment might be required to achieve tumor control. Therefore, blocking the interaction between inhibitory Siglecs on immune cells and sialo-ligands on cancer cells may overcome immune checkpoint escape and boost cancer immunotherapy.

ST3GAL1 and ST3GAL4 are upregulated in patients with pancreatic ductal adenocarcinoma (PDAC) [19]. The ahpha 2,3 sialylated glycans derived from PDAC cells modulate the differentiation of monocytes to immune-suppressive tumor-associated macrophages (TAMs) through the activation of Siglec-7 and -9 [19]. TAMs account for 20–40 % of infiltrating lymphocytes in the TME of HCC [43]. It has been shown that CD68^+^ TAMs express high levels of Siglec-7 and -9 in primary high-grade serous ovarian cancer (HGSC) specimens [44]. Moreover, blood-derived monocytes or macrophages express high levels of Siglec-7 and -9 [19]. Our finding that Siglec-7 and -9 ligands are upregulated in sorafenib-resistant Huh7 and HepG2 cells (Fig. 2) suggests a mechanism of immune suppression mediated by monocytes/macrophages in addition to NK cells, through increased Siglec-7/9 ligands in drug-resistant HCC in the TME.

Chronic lymphocytic leukemia (CLL) B cells express high levels of Siglec-7 ligands. *ST6GALNAC4* knockdown in CLL B cells decreased the expression of disialyl T (Neu5Acα2-3Galβ1-3[Neu5Acα2-6]GalNAcα1-) glycans, which was correlated with reduced Siglec-7 chimera binding [45]. The expression of *ST6GALNAC4* was not upregulated in HCC cells after chronic sorafenib treatment; however, the expression of *ST3GAL1* and *ST6GALNAC5* was significantly induced in both HCC sorafenib-resistant cell lines. Whether ST6GALNAC5 is correlated with the expression of Siglec ligands in HCC awaits future investigation.

In this study, we found that high *ST3GAL1* expression was associated with poor clinical outcomes in patients with stage 1-2 HCC and in sorafenib-resistant HCC cell lines. All tested HCC cell lines expressed Siglec-7 ligands at varying levels; however, Siglec-9 ligands were detected highly in Huh7 and sorafenib-resistant Huh7 or HepG2 cells. Silencing *ST3GAL1* in both parental and sorafenib-resistant HCC cells reduced the expression of Siglec-7 ligands and some Siglec-9 ligands.

This differential effect may reflect differences in glycan structures preferentially recognized by Siglec-7 and Siglec-9, as well as cell line-specific glycosyltransferase expression profiles. Siglec-7 primarily recognizes α2,3-sialylated glycans commonly generated by ST3GAL1/2 and α2,6-sialylated glycans produced by members of the ST6GALNAC family [20, 45]. In contrast, Siglec-9 exhibits broader glycan specificity and can preferentially bind more complex glycan motifs synthesized by alternative sialyltransferases, including ST3GAL4, ST6GAL1, ST8SIA, as well as ST6GALNAC family members [20]. Our qPCR analysis of the sialyltransferase family showed that several sialyltransferases are expressed at higher levels in Huh7 than in other tested HCC cell lines (Supplementary Fig. S8). The distinct glycosylation program in the Huh7 cell line is consistent with its selective expression of Siglec-9 ligands. Together, these findings highlight the heterogeneity of tumor-associated sialylation and Siglec ligand expression in HCC.

ST3GAL1 participates in the biosynthesis of α2,3-sialylated O-glycans, and its inhibition may influence additional glycan structures beyond those directly involved in Siglec-7 ligand formation. Our previous studies showed that proteins such as NRP1 [46], GFRA1 [47], and CD55 [48] carry O-linked sialoglycans including disialyl-T, which is recognized by Siglec-7, suggesting that ST3GAL1 may regulate multiple sialylated glycoproteins and glycan structures. Therefore, potential off-target effects of *ST3GAL1* silencing on other glycan-dependent pathways should be considered.

Notably, ST3GAL1-mediated sialylation of CD55 impedes C3 deposition and reduces complement-dependent cytotoxicity (CDC) in cancer cells [48]. Thus, ST3GAL1-mediated O-linked sialylated CD55 may function as an immune checkpoint that protects tumor cells from immune attack. Silencing *ST3GAL1* may therefore enhance both NK cell-mediated ADCC and complement-dependent lysis, potentially improving therapeutic efficacy.

Chronic HBV infection accounts for approximately 60 % of HCC cases in Asia and Africa, whereas chronic HCV infection is a major etiology of HCC in North America, Europe, and Japan [49]. The mean total serum concentration of sialic acid in patients with chronic HCV infection has been reported to be significantly higher than that in patients with chronic HBV infection [50]. Notably, an increased prevalence of α2-3-linked sialic acid in serum alpha-fetoprotein (AFP) has been observed in patients with HCV-induced cirrhosis (HCV-LC), whereas increased α2-6-linked sialic acid in AFP was detected in patients with chronic hepatitis B (HBV-CH) compared with healthy individuals [51].

Consistent with these findings, the increased expression of *ST3GAL1* in HCC tissues from patients with HCV infection (Supplementary Fig. S4e) may reflect enhanced α2-3 sialylation in the HCV-associated disease context. However, whether ST3GAL1 upregulation is directly driven by viral factors or secondary to chronic inflammation-mediated remodeling of the hepatic microenvironment remains unclear. Further studies will be required to clarify the underlying mechanisms.

In conclusion, we demonstrated that the upregulation of ST3GAL1 correlated with poor relapse free survival in patients with stage 1–2 HCC. Acquired sorafenib resistance upregulates the expression of ST3GAL1 and the surface expression of Siglec-7/9 ligands, protecting tumor cells from immune surveillance. Downregulation of ST3GAL1 expression results in increased NK-mediated cytotoxicity and ADCC against tumor cells. These findings suggest that ST3GAL1 silencing may be a promising strategy for immune checkpoint blockade by down-regulating Siglec-7 ligands to enhance NK-mediated cytotoxicity and ADCC.

### Limitations of the study

ST3GAL1 upregulation increases the display of Siglec-7 ligands on the cell surface. However, the specific glycoproteins that carry these sialylated structures have not yet been identified in HCC, representing an important knowledge gap in understanding sialylation-driven immune evasion. Recent studies have identified podocalyxin and MUC13 as Siglec-7 ligands in colon cancer cells [52]. Upregulation of such glycoproteins together with sialyltransferases in tumors may enhance cancer cell invasion and promote immune evasion. Future glycoproteomic analyses will be necessary to identify the specific glycoprotein carriers of Siglec-7 ligands in HCC which may reveal novel immunotherapeutic targets to improve patient outcomes.

## Supplementary Information

Below is the link to the electronic supplementary material.

Supplementary file1 (DOCX 3725 KB)

## Data Availability

The data that support the findings of this study are available from the corresponding author, (AY), upon request.

## Abbreviations

ADCC: antibody-dependent cellular cytotoxicity
HCC: hepatocellular carcinoma
ST3GAL1: ST3 beta-galactoside alpha-2,3-sialyltransferase 1
