# Supplementary material for: Targeting ST3GAL1 to downregulate ligands for the glycoimmune checkpoint Siglec-7 and reverse immune escape in hepatocellular carcinoma
Source: Cancer Immunol Immunother. 2026 Apr 10;75(5):140. doi: 10.1007/s00262-026-04388-x (PMC13069034; doi:10.1007/s00262-026-04388-x)
Supplement: Supplementary file 1 — Supplementary file1 (DOCX 3725 KB) [file 262_2026_4388_MOESM1_ESM.docx]

Supplementary data for

**Targeting ST3GAL1 to downregulate ligands for the glycoimmune checkpoint Siglec-7 and reverse immune escape in hepatocellular carcinoma**

**File list:**

**Fig. S1.** qPCR analysis of the *ST3GAL1* transcript level in HCC cell lines, control pVoid or *shST3GAL1* transfected HCC cells using *GAPDH* expression as an internal control.

**Fig. S2.** Long-term sorafenib treatment induced the expression of Siglec-7/9 ligands.

**Fig. S3.** WB and FACS analysis of EGFR expression.

**Fig. S4.** Correlation of clinicopathological parameters with the expression levels of ST3GAL1 in HCC patients.

**Fig. S5.** Analysis of survival outcomes in patients with HCC using TCGA database.

**Fig. S6.** Elevated *ST3GAL1* expression is associated with poorer immunotherapy response across a pan-cancer cohort.

**Fig. S7.** qPCR analysis of the *PD-L1* and *VEGFA* transcript level in sorafenib-resistant HCC cell lines.

**Fig. S8.** Heatmap of sialyltransferase mRNA levels in HCC cell lines assessed by qPCR analysis.

**Table S1.** Antibodies and lectin used in this study.

**Table S2.** Clinical and pathological characteristics of 166 HCC patients.

**Table S3.** qPCR primers used in this study.

**Fig. S1.** qPCR analysis of the *ST3GAL1* transcript level in different HCC cell lines (a), or in control pVoid or *shST3GAL1* transfected HCC cells (b) using *GAPDH* expression as an internal control. The data are presented as the mean ± standard deviation, based on representative results from three independent experiments (n = 3).

**
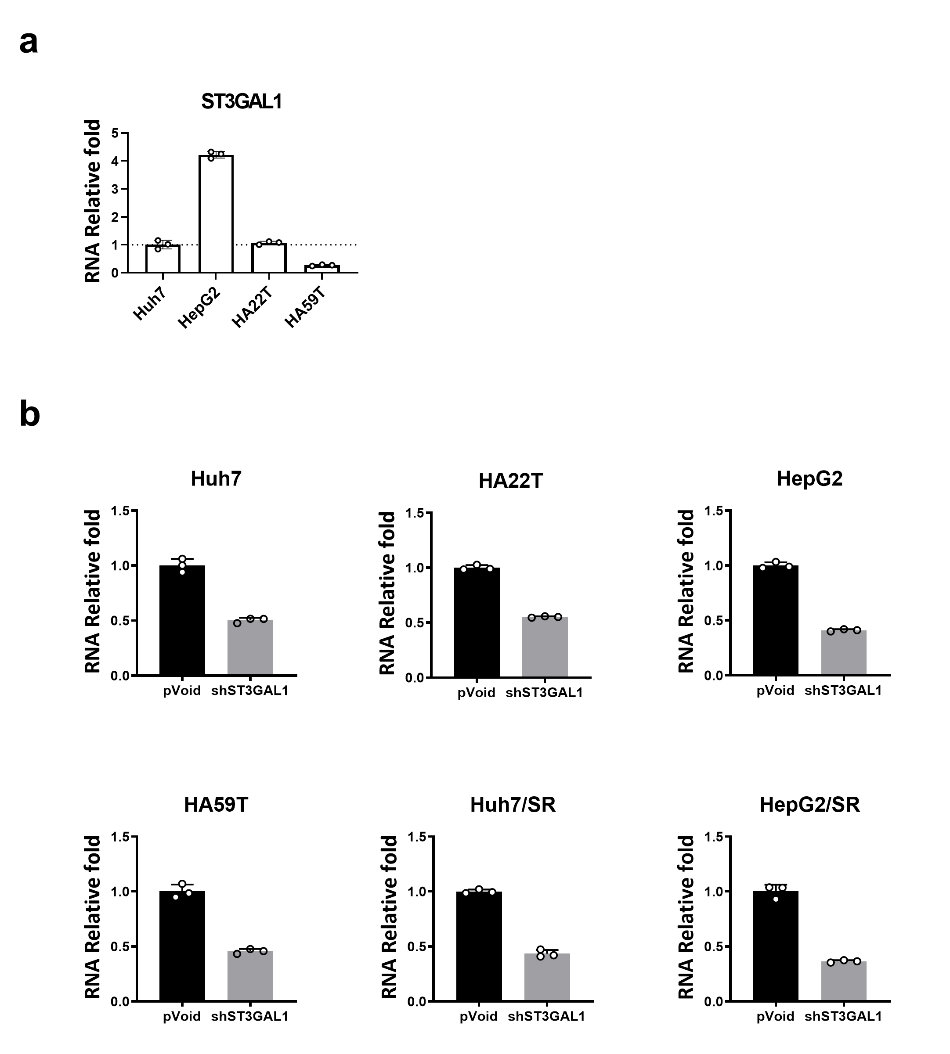
**

**Fig. S2.** Long-term sorafenib treatment induced the expression of Siglec-7/9 ligands. Cells were stained with recombinant Siglec-Fc chimeras, detected with goat anti-human IgG-AF488 and analyzed by flow cytometry. (a) Sorafenib-resistant cells were stained with recombinant Siglec-Fc chimeras and analyzed by flow cytometry. (b) Flow cytometric histograms showing Siglec-7 or Siglec-9 ligand staining in control (pVoid) and *ST3GAL1*-silenced cells generated by *shST3GAL1* transfection. (c) Control pVoid or *shST3GAL1* transfected sorafenib-resistant cells were stained with recombinant Siglec-Fc chimeras and analyzed by flow cytometry. Representative overlay histograms from three independent experiments (n=3) are shown. WT: wild type; RA: Siglec-7/9 Arg120 to Ala mutant; SR: sorafenib-resistant; MFI: mean fluorescence intensity.

**
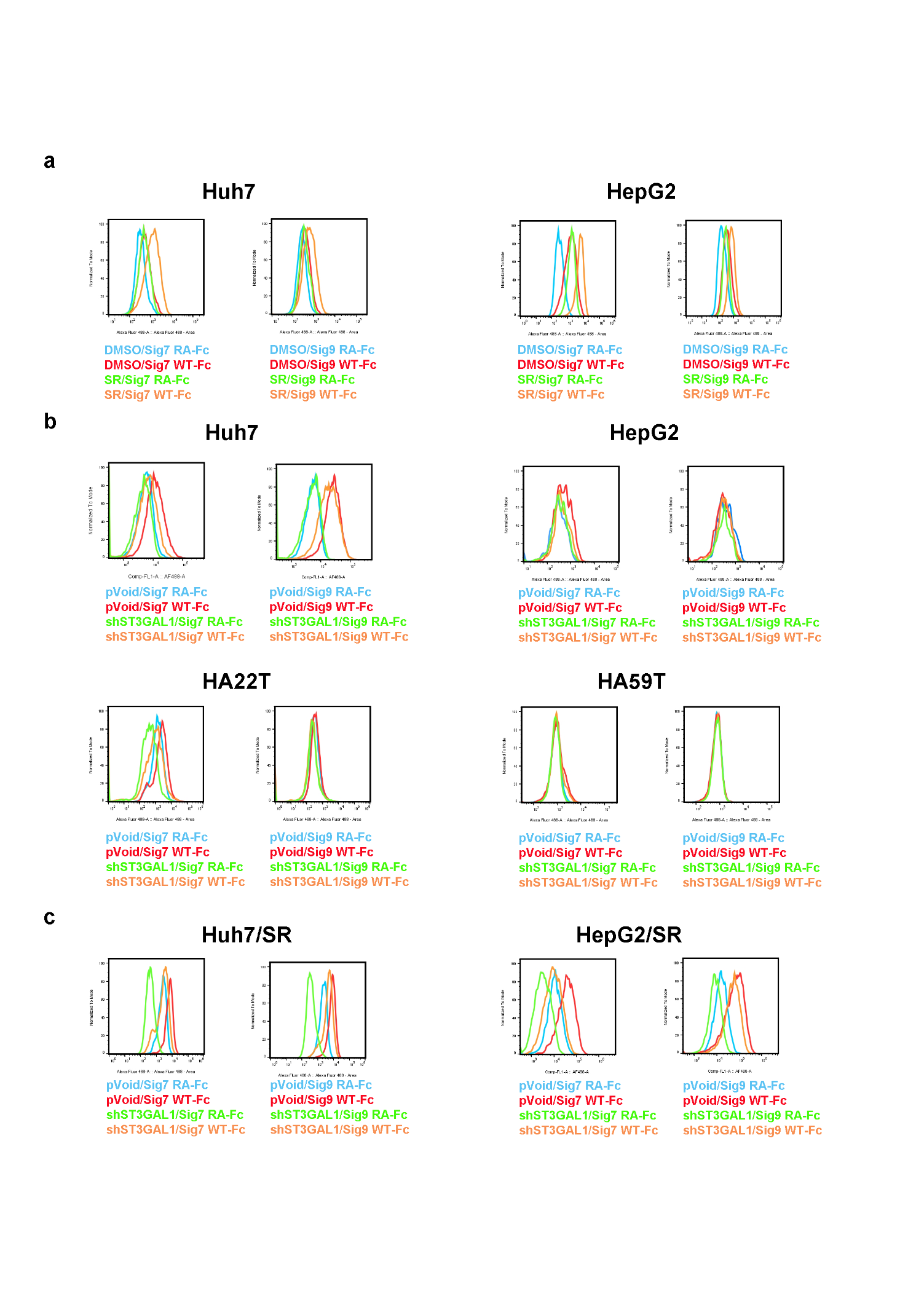
**

**Fig. S3.** WB and FACS analysis of EGFR expression. WB analysis of EGFR in (a) HCC cell lines; (b) control pVoid or *shST3GAL1* transfected cells or sorafenib-resistant cells. (a) The expression of EGFR was normalized to GAPDH, and the EGFR levels in each cell line were subsequently normalized to those expressed in HA22T. (b) The expression of EGFR was normalized to GAPDH or Tubulin. The EGFR levels in *shST3GAL1*-transfected cells or sorafenib-resistant cells were then normalized to the respective control cells for each cell line. (c) Histograms showing the binding of cetuximab to HCC cells. The blue lines represent unstained controls. Red lines represent isotype Ab stained control. The orange lines represent cetuximab binding, followed by goat anti-human IgG-AF488 staining. The MFI value was displayed alongside the histogram. p: pVoid; sh: *shST3GAL1*; D: DMSO; SR: sorafenib-resistant; MFI: mean fluorescence intensity.


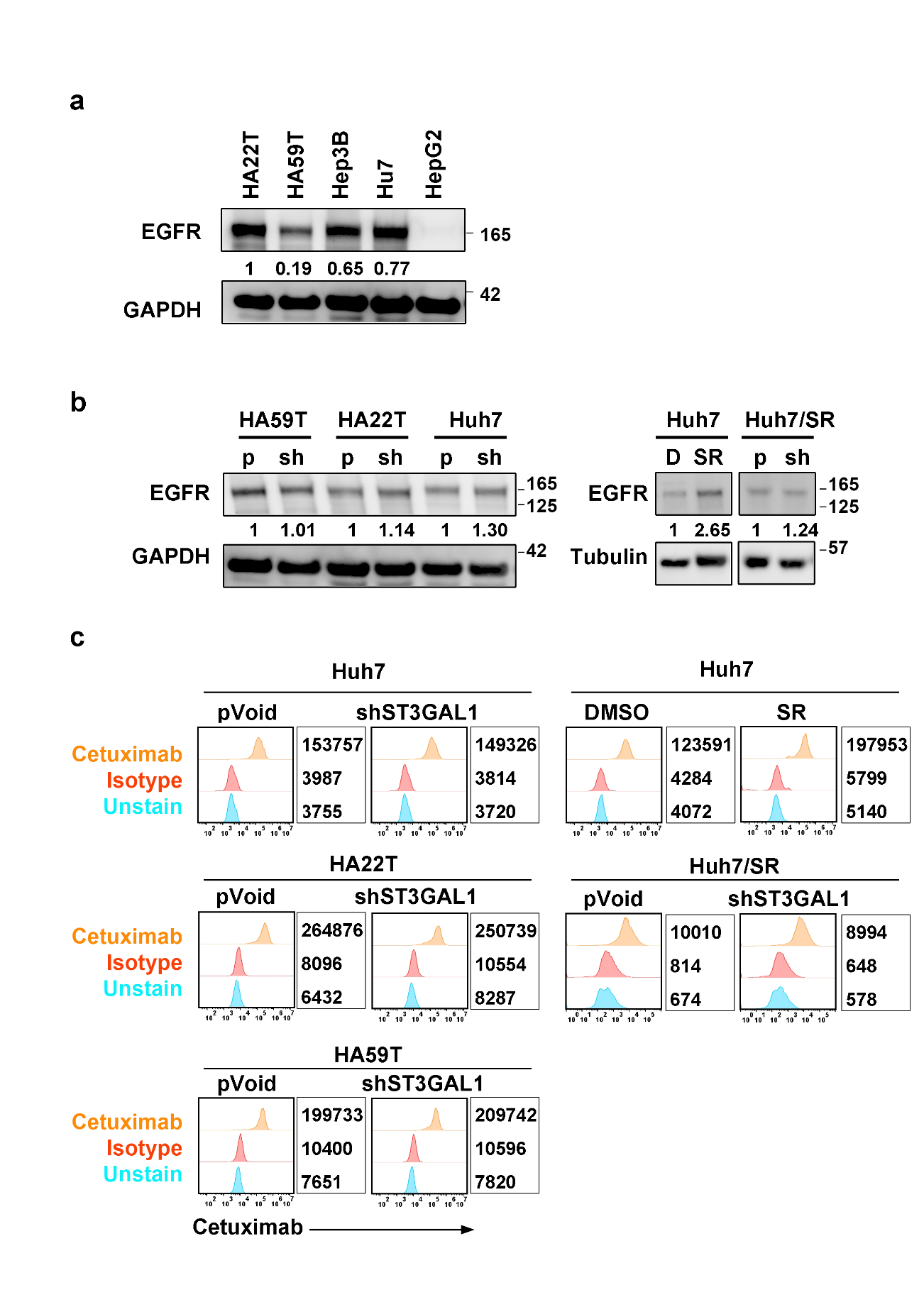


**Fig. S****4.** Correlation of clinicopathological parameters with the expression levels of *ST3GAL1* in HCC patients. The mRNA expression levels of *ST3GAL1* normalized to those of *GADPH* and expressed as -delta Ct data were correlated with age (a), sex (b), drinking history (c), smoking history (d), hepatitis virus infection status (e), tumor size (f), tumor grade (g), tumor stage (h) and cirrhosis status (i) in HCC patients. A total of 166 patients were included in the study. However, data were missing for the following parameters: drinking history (1 patient), smoking history (1 patient), and hepatitis virus infection status (1 patient). Statistical analysis was conducted using the Mann-Whitney U test. Ct: cycle threshold.

**Fig. S5.** Analysis of survival outcomes in patients with HCC using TCGA database. Kaplan-Meier analysis of disease-free survival (DFS) according to *ST3GAL1* mRNA expression in HCC using the TCGA Liver Hepatocellular Carcinoma cohort (TCGA GDC, 2025) accessed through cBioPortal. Gene expression levels were analyzed using mRNA expression (FPKM z-scores). (a) In the all-stage cohort, patients with high *ST3GAL1* expression (n = 31) exhibited significantly shorter DFS compared with those with low *ST3GAL1* expression (n = 284) (log-rank test, P = 0.03). (b) In the early-stage subgroup, high *ST3GAL1* expression (n = 27) was also associated with poorer DFS compared with low *ST3GAL1* expression (n = 204) (log-rank test, P = 0.03).

**a b**

**Fig. S6**. Elevated *ST3GAL1* expression is associated with poorer immunotherapy response across a pan-cancer cohort. Kaplan-Meier survival curves were generated using the Kaplan-Meier Plotter immunotherapy database, which integrates gene expression profiles and clinical outcome data from patients with solid tumors treated with immune checkpoint inhibitors. *ST3GAL1* was selected as the gene of interest, and progression-free survival (PFS) was used as the survival endpoint. Only pretreatment tumor samples were included in the analysis. Patients were stratified into high- and low-expression groups using the automatically selected optimal cutoff based on percentile distribution. Subgroup analyses were performed according to treatment with anti-PD-1 (a), anti-PD-L1 (b), or anti-CTLA-4 (c) therapies. Hazard ratios (HRs) and *P* values were calculated using the log-rank test.

**
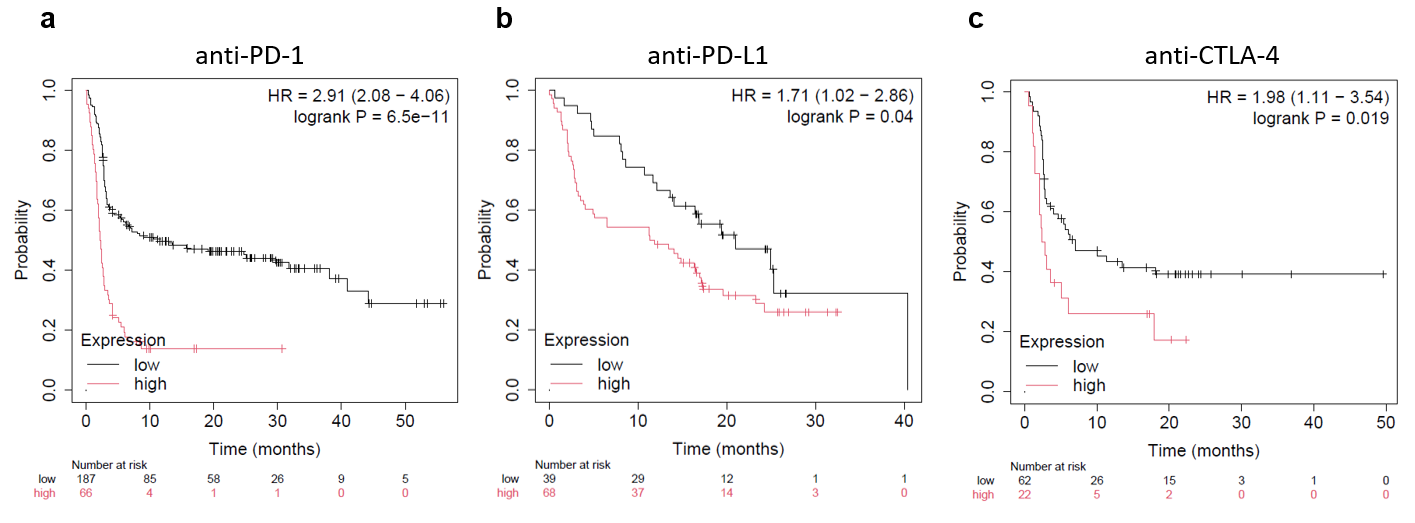
**

**Fig. S7.** qPCR analysis of the (a) *PD-L1* and (b) *VEGFA* transcript level in sorafenib-resistant HCC cell lines, or in control pVoid or *shST3GAL1* transfected sorafenib-resistant HCC cells using *GAPDH* expression as an internal control. The *PD-L1* or *VEGAF* levels in sorafenib-resistant cells or *shST3GAL1*-transfected cells then normalized to the respective control cells for each cell line. The data are presented as the mean ± standard deviation, based on representative results from three independent experiments (n = 3). SR: sorafenib-resistant.


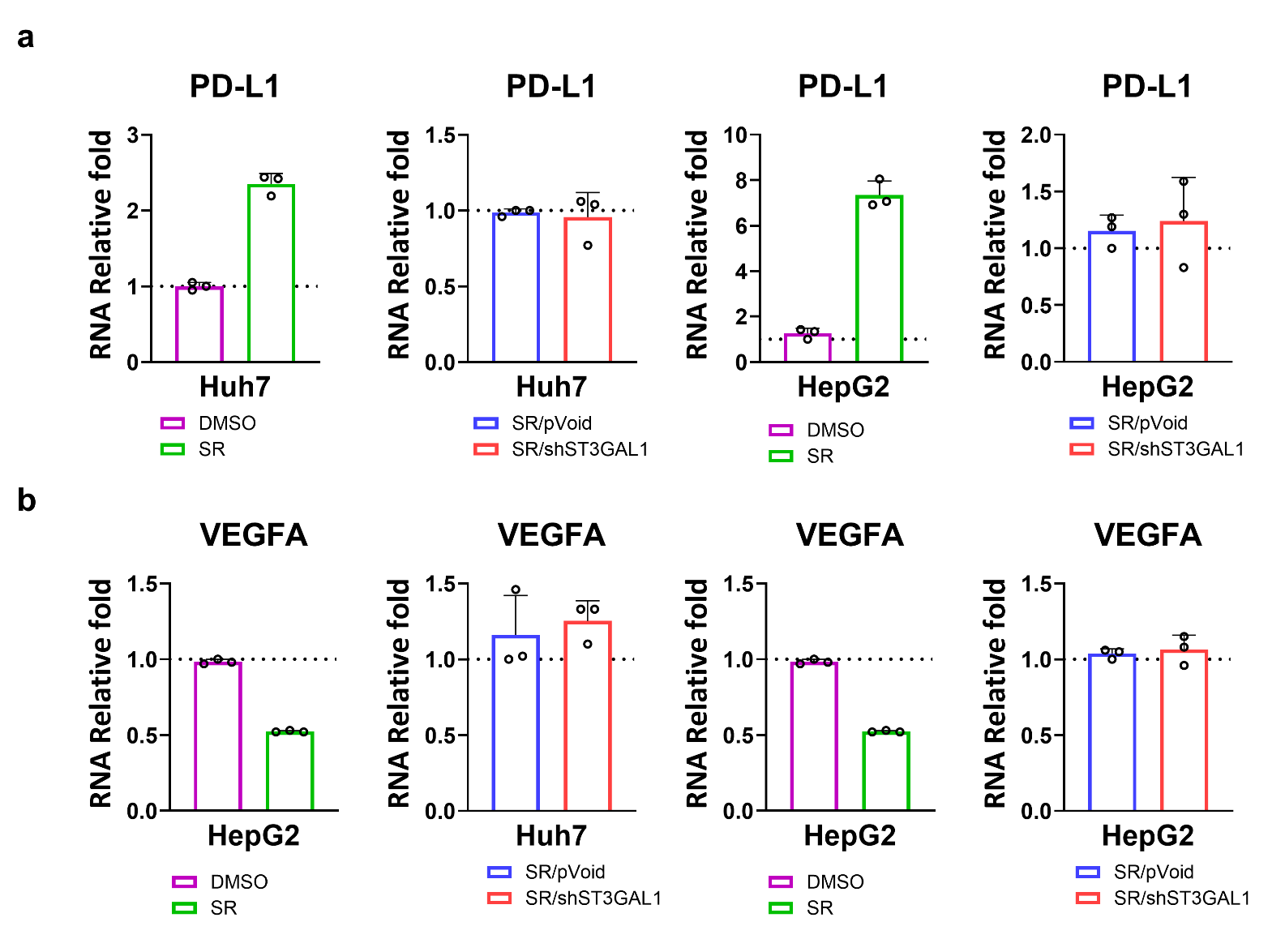


**Fig. S8.** Heatmap of sialyltransferase mRNA levels in HCC cell lines assessed by qPCR analysis. The mRNA expression levels of each sialyltransferase were normalized to GAPDH and presented as −ΔCt values to improve comparability. Ct, cycle threshold.

**
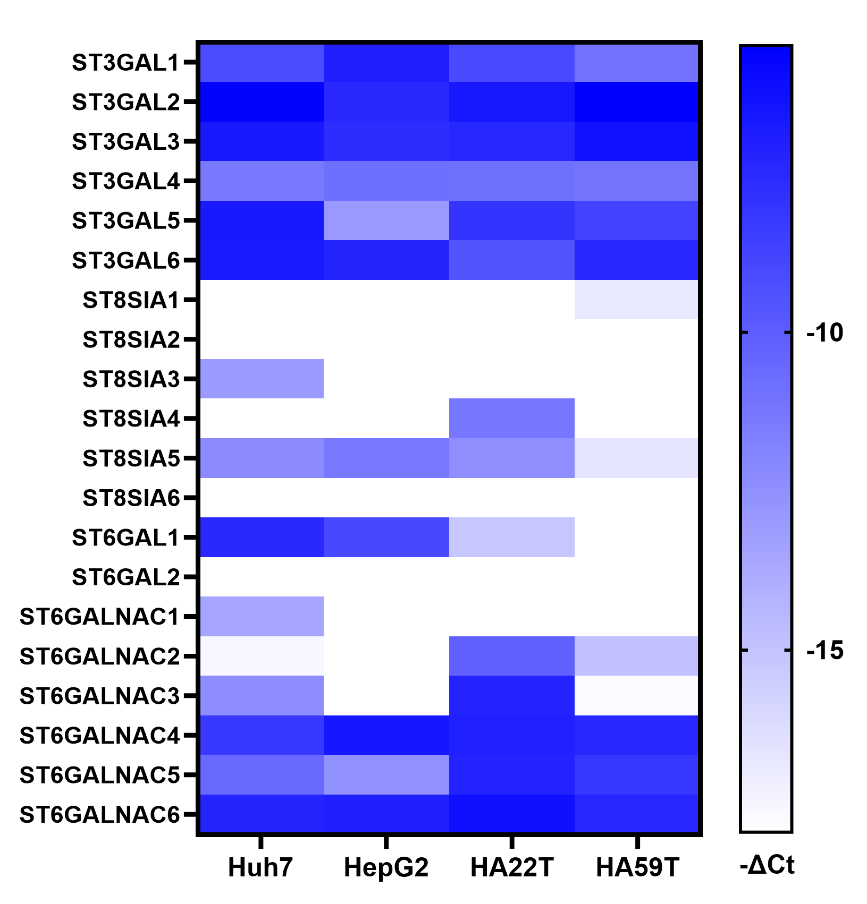
**

**Table S1.** Antibodies and lectin used in this study

| Markers | Catalog no | RRID no |
| --- | --- | --- |
| CD56-BV421 | BioLegend Cat# 318328 | RRID:AB_11218798 |
| CD56-FITC | BioLegend Cat# 318304 | RRID: AB_604100 |
| CD3-PE | BioLegend Cat# 300408 | RRID:AB_314062 |
| CD3-APC | BioLegend Cat# 300439 | RRID:AB_2562045 |
| CD16-AF700 | BioLegend Cat# 302026 | RRID:AB_2278418 |
| CD16-APC-Cy7 | BioLegend Cat# 302017 | RRID:AB_314217 |
| CD107a-BV711 | Biolegend Cat# 328640 | RRID:AB_2565840 |
| IFN-g-PE/Cy7 | Biolegend Cat# 502528 | RRID:AB_2123323 |
| IFN-g-BV711 | Biolegend Cat# 502540 | RRID:AB_2563506 |
| CD45-APC | Biolegend Cat# 304037 | RRID:AB_2562049 |
| Siglec-7-APC | BioLegend Cat# 339206 | RRID:AB_2565239 |
| Siglec-7-PE | BioLegend Cat# 339203 | RRID:AB_1501164 |
| Siglec-9-FITC | BioLegend Cat# 351512 | RRID:AB_2728327 |
| EGFR | Cell Signaling Cat# 4267S | RRID:AB_2246311 |
| GAPDH | GeneTex Cat# GTX100118 | RRID:AB_1080976 |
| Goat anti-human IgG-AF488 | Jackson ImmunoResearch Cat# 109-545-003 | RRID:AB_2337831 |
| Alkaline phosphatase-conjugated goat anti-rabbit IgG antibody | Jackson ImmunoResearch Labs Cat# 111-055-144 | RRID:AB_2337953 |
| Control IgG | Jackson ImmunoResearch Labs Cat# 009-000-003 | RRID:AB_2337043 |
| Alkaline phosphatase-conjugated streptavidin | Jackson ImmunoResearch Cat# 016-050-084 |  |
| Cetuximab | Erbitux |  |
| PNA-Biotinylated | VectorLabs Cat# B1075 |  |

**Table S2.** Clinical and pathological characteristics of 166 HCC patients. The average age of the HCC patients was 60.1 years, with a standard deviation of 12.7 years (range, 21–83 years). Within this group, 74.7 % of the patients were male, 54.2 % tested positive for hepatitis B virus (HBV), and 31.9 % were positive for hepatitis C virus (HCV). The mean tumor size was 6.3 cm, ranging from 1 cm to 20 cm, with 51.2 % of patients having tumors larger than 5 cm. Furthermore, 96 patients (61.7 %) had low-grade tumors, and 57 patients (34.3 %) were diagnosed with liver cirrhosis. According to the Tumor-Node-Metastasis (TNM) staging system, 107 patients (64.5 %) were classified as having stage I or II disease. The median follow-up durations for relapse-free and overall survival were 24.8 months (ranging from 0.7 to 163.8 months) and 67.4 months (ranging from 0.7 to 163.8 months) respectively. At the time of this study, 54.8 % (91 out of 166) of patients had died.

| **Characteristics** | **N (%)** |
| --- | --- |
| **Age (Mean±SD and range) (years)** | 60.1 ± 12.7 (21-83) |
| **Gender** | |
| Male | 124 (74.7%) |
| Female | 42 (25.3%) |
| **Drinking history^a^** | |
| Yes | 57 (34.5%) |
| No | 108 (65.5%) |
| **Smoking history^b^** |  |
| Yes | 81 (49.1%) |
| No | 84 (50.9%) |
| **Virus infection** | |
| None | 22 (13.4%) |
| HBV | 90 (54.2%) |
| HCV | 53 (31.9%) |
| HBV+HCV | 1 (0.6%) |
| **Tumor size (Mean±SD and range) (cm)** | 6.3 ± 4.1 (1-20) |
| ≦5 | 81 (48.8%) |
| >5 | 85 (51.2%) |
| **Edmondson Grade** | |
| 1+2 | 96 (67.1%) |
| 3+4 | 47 (32.9%) |
| **TNM stage** | |
| I+II | 107 (64.5%) |
| III+IV | 59 (35.5%) |
| **Cirrhosis** | |
| No | 109 (65.7%) |
| Yes | 57 (34.3%) |
| **Relapse** | |
| No | 58 (34.9%) |
| Yes | 108 (65.1%) |
| RFS duration median (range) | 24.8 (0.7–163.8 months) |
| **Outcomes** | |
| Alive | 75 (45.2%) |
| Dead | 91 (54.8%) |
| OS duration median (range) | 67.4 (0.7–163.8 months) |

^a^ One patient had no drinking history data. ^b^ One patient had no smoking history data. RFS: relapse free survival; OS: overall survival.

**Table S3.** qPCR primers used in this study

Name SYBR primer sequences

*ST3GAL1* Forward primer 5’-TTGGGTCAGGACAATTCCAT-3’

Reverse primer 5’-CAGCTTGATGAAGGCACAGA-3’

*GAPDH* Forward primer 5’-CTGCTCCTCCTGTTCGACAGT-3’

Reverse primer 5’-ACCTTCCCCATGGTGTCTGA-3’

Name Taqman probes

*ST3GAL1* Hs00161688_m1

*ST3GAL2* Hs00911835_m1

*ST3GAL3* Hs00544033_m1

*ST3GAL4* Hs00272170_m1

*ST3GAL5* Hs00187405_m1

*ST3GAL6* Hs00196086_m1

*ST6GAL1* Hs00949382_m1

*ST6GAL2* Hs00383641_m1

*ST6GALNAC1* Hs00300842_m1

*ST6GALNAC2* Hs00197670_m1

*ST6GALNAC3* Hs00541761_m1

*ST6GALNAC4* Hs01082656_m1

*ST6GALNAC5* Hs05018504_s1

*ST6GALNAC6* Hs00203739_m1

*ST8SIA1* Hs00268157_m1

*ST8SIA2* Hs00916611_m1

*ST8SIA3* Hs01026908_m1

*ST8SIA4* Hs00379924_m1

*ST8SIA5* Hs00203298_m1

*ST8SIA6* Hs02341869_g1

*GAPDH* Hs99999905_m1
